# Technological Innovations and the Translational Path of Kidney Organoids

**DOI:** 10.3390/biomedicines14020327

**Published:** 2026-01-31

**Authors:** Anni Li, Zhonglin Chai, Karin Jandeleit-Dahm, Jay C. Jha

**Affiliations:** 1 Department of Endocrine and Metabolic Diseases, School of Medicine, Southeast University, Nanjing 210032, China; 2 Department of Diabetes, School of Translational Medicine, Monash University, Melbourne, VIC 3004, Australia

**Keywords:** kidney organoids, human pluripotent stem cells, disease modeling, drug screening, regenerative medicine

## Abstract

Kidney organoids, as three-dimensional microstructures derived from human pluripotent stem cells or adult stem cells, precisely simulate the cellular heterogeneity, spatial conformation, and some physiological functions of human kidney units in vitro. Kidney organoids are three-dimensional microstructures derived from human pluripotent stem cells (hPSCs). They precisely simulate the cellular heterogeneity, spatial conformation, and key physiological functions of human kidney units in vitro. This technology, by replicating the interaction network between the glomerulus and renal tubules, provides an unprecedented window for observing the dynamic development and pathological processes of human kidneys. This technology replicates the interaction network between the glomerulus and renal tubules. It thereby provides an unprecedented window into human kidney development and disease. Based on the strong similarity between organoids and native organs, as well as the human genetic information they carry, both iPSC-derived and patient-specific organoids have demonstrated significant value in kidney disease modeling, drug toxicity testing, and the development of regenerative treatment strategies. This review systematically elucidates the key advancements in the field of kidney organoids, including optimized strategies for stem cell-directed differentiation, innovations in culture systems driven by biomaterials engineering, technological breakthroughs in disease model construction, and applications of organoids in drug screening platforms and regenerative medicine. Additionally, it analyzes translational challenges such as the lack of vascularization, insufficient functional maturity, and obstacles in standardized production. These insights will deepen the understanding of kidney pathological mechanisms and propel organoid technology towards substantial clinical therapeutic applications. This review summarizes how convergent technologies in stem cell biology and bioengineering aim to bridge this functional gap. We examine the use of advanced organoids in disease modeling and drug discovery. We also highlight their current limitations. Our focus is on the core translational bottlenecks: vascularization, long-term maturation, and scalable production. Overcoming these hurdles is essential to transform kidney organoids from a research tool into a platform for precision medicine and regenerative therapy.

## 1. Introduction

The global burden of kidney disease continues to worsen, with the combined burden of diabetes and kidney disease increasing by 25% since 1990 [1]. Epidemiological data show that approximately 20% of hypertensive patients develop kidney disease, while up to 40% of diabetic patients suffer from kidney damage, with diabetic kidney disease (DKD) becoming one of the leading causes of end-stage renal failure [2,3]. In China, about one-third of adult diabetic patients suffer from chronic kidney disease, a phenomenon closely linked to genetic backgrounds, environmental factors, and unequal distribution of medical resources [4,5]. It also imposes a heavy economic burden globally [6,7,8]. Notably, the mortality rate associated with chronic kidney disease has steadily increased over the past 30 years, highlighting the urgency of its public health threat [9,10].

The traditional research models expose clear limitations in addressing this challenge. Two-dimensional cell cultures fail to replicate the kidney’s three-dimensional structure and intercellular interactions. Animal models, meanwhile, present species differences that hinder the translation of drug responses and disease phenotypes to humans [11,12]. Although dialysis therapy can substitute part of the kidney filtration function, it cannot replicate key physiological activities such as endocrine regulation, and the patient’s quality of life is significantly restricted [13]. Organ transplantation faces the persistent dilemma of donor shortages. Globally, only about 5.3% of end-stage renal disease patients receive a transplant annually. The remaining patients are forced to rely on lifelong dialysis [13,14]. Therefore, to address the aforementioned issues, finding new, more comprehensive experimental models and more effective clinical alternatives has become an urgent priority.

The rise of organoid technology marks a revolution in kidney research paradigms. These three-dimensional micro-organs, derived from pluripotent or adult stem cells, simulate the process of embryonic kidney development, forming self-organizing structures that include glomeruli, renal tubules, and interstitial cells. The core breakthrough lies in the reproduction of the spatial conformation and cellular heterogeneity of kidney units, providing a platform with higher physiological relevance for disease modeling [15,16]. In recent years, the application of human-expanded potential stem cells (hEPSC) has further enhanced the differentiation efficiency of kidney organoids. For example, the construction of the SIX2 reporter gene system using CRISPR-Cas9 technology has enabled real-time tracking of kidney progenitor cell differentiation [17], revealing the key regulatory role of the Hippo-YAP signaling pathway in organoid maturation [18,19]. At the functional level, transplantation experiments have confirmed that kidney organoids with some degree of microvascularization can partially restore kidney function [20]. For instance, transplanting embryonic kidney tissue containing 50,000 kidney units has enabled the survival of kidney-deficient rats for over 30 days, providing a proof-of-concept for regenerative medicine [21].

This review aims to critically discuss the technological breakthroughs and translational challenges in the field of kidney organoids. The focus is on analyzing the optimization strategies for stem cell-directed differentiation protocols, including innovations in biomaterial scaffold design, mechanical force application, and vascular network construction. It addresses a central question. How can kidney organoid technology achieve true functional and translational relevance? First, we analyzed key advancements in stem cell differentiation and culture systems. These innovations target the core bottlenecks of functional maturity and vascularization. Next, we evaluated how these advanced models are applied to study disease mechanisms and improve drug development. We assessed both their predictive power and current constraints. Finally, we synthesized the persistent obstacles in standardization and scalability from a clinical perspective. This review provides a roadmap for researchers aiming to harness kidney organoids to meet unmet clinical needs in nephrology.

## 2. Key Technological Advancements in Kidney Organoids

### 2.1. Precise Regulation of Stem Cell Differentiation Strategies

The directed differentiation of human pluripotent stem cells (hPSCs) into kidney organoid cultures has brought revolutionary breakthroughs. By accurately simulating development, optimizing differentiation strategies, constructing three-dimensional microenvironments, enhancing complexity and maturity, integrating vascularization, and incorporating gene editing along with pushing for standardization, a high degree of simulation of human kidney structure has been achieved in vitro to some extent [22,23,24].

Building on the pioneering work of Taguchi et al., the establishment of hPSC-derived kidney organoids begins with the formation of embryoid body aggregates, followed by the induction of posterior mesoderm via activin A and CHIR99021 (Wnt agonist). This induces differentiation into metanephric mesenchyme (MM) cells, which, when co-cultured with mouse embryonic spinal cord tissue, forms organoids with nephron-like structures [25]. However, there are still problems such as impure cell types and immature functions. Subsequent studies have been optimized around the following key dimensions. Subsequently, more studies have enriched the structure of kidney organoids. For example, Yanofsky et al. demonstrated that ANG II inhibits tissue development in the early stages of iPSC-derived kidney organoid development, but in the mid-stage, ANG II intervention promotes the formation of kidney-derived mesenchyme, ureteric bud tips, and podocytes [26]. Building on previous studies, Vanslambrouck et al. proposed a strategy of extending Wnt signaling activation time and adding FGF9 in a temporal manner, significantly improving the maturity and functionality of proximal tubular cells. The induced kidney organoids exhibited albumin absorption and organic cation transport capabilities, triggered mesenchymal–epithelial transition in NPCs (Nephron Progenitor Cells), and initiated nephron patterning [27]. This innovation primarily addresses the challenge of proximal tubule maturation. Huang et al. achieved long-term two-dimensional expansion of kidney progenitor cells through small molecule regulation of p38 and YAP signaling pathways, solving the issue of cellular heterogeneity in traditional three-dimensional cultures, and successfully generating high-purity podocyte organoids [28]. Compared to sequential additions, this technology improved podocyte purity. Furthermore, the integration of 3D bioprinting and microfluidic technologies have further enhanced the structural complexity of kidney organoids. For instance, a team from Harvard used 3D-printed chips to apply fluid shear stress, promoting the formation of perfusable vascular networks within the organoids and simulating the glomerular filtration microenvironment [29]. Thus, organoid technology of the kidney is moving from simple morphological imitation to functional oriented precision design and engineering construction.

### 2.2. Engineering Innovation of Culture Systems

Innovations in culture systems have further optimized the structural development of kidney organoids. These optimizations focus on the extracellular matrix microenvironment and vascularization strategies. For instance, during the culture process, synthetic hydrogels have gradually replaced animal-derived matrix gels [23,30,31] (such as Matrigel), significantly enhancing the spatial organization precision of kidney organoids due to the controllable mechanical properties and composition of these hydrogels [32]. For example, Clerkin et al. used semi-synthetic methacryloyl gelatin (GelMA) hydrogels to differentiate hiPSC-derived kidney organoids. Their research found that kidney organoids derived in this hydrogel were closer to the stiffness of adult kidneys (~5000–10,000 Pa. Pascal, a unit of hydrogel stiffness) compared to those cultured in traditional matrix gels, showing improved podocyte maturation and upregulation of kidney vesicle-related genes in the early stages of encapsulation when compared to organoids derived from softer hydrogels (~400 Pa) [33].

Vascularization strategies have successfully induced the formation of interconnected vascular lumens within organoids through co-culturing human umbilical vein endothelial cells or adding vascular endothelial growth factors, combined with mechanical stimulation from bioreactors. For example, Morizane et al. confirmed that fluid shear stress can promote endothelial cell migration and encase the renal tubule structure [29]. Furthermore, Garreta et al. used a method involving the transplantation of chick chorioallantoic membranes to facilitate the efficient generation of kidney vesicles and nephron structures, which helped address vascularization issues to some extent [34]. Insufficient vascular network development is another core bottleneck. The construction of a vascular network is particularly critical for organs like the kidney, which have rich blood supply. Currently, only a few organoids can form functional microvessels, and they lack integration with larger blood vessels, potentially leading to delayed glomerular development and low filtration efficiency. There have been preliminary studies on vascularization following kidney organoid transplantation, such as one in which the researchers effectively induced and studied the stable development of vascular networks in kidney organoids through transplantation into the body cavity of chick embryos [35]. Homan et al.’s research found that kidney organoids cultured on a microfluidic chip under fluid flow exhibited more mature podocytes and renal tubule compartments, with enhanced cell polarity and increased expression of adult genes [29]. At the same time, research teams have developed protocols to effectively differentiate hPSCs into segment-patterned 3D kidneys and organoids related to newly developed vascular networks, which have provided a reliable platform for large-scale drug testing to some extent [36]. Although new hPSC differentiation protocols have promoted vascular network development, kidney organoids still generally face the challenge of low rates of functional microvascular formation and the absence of large blood vessel integration. Current approaches (such as fluid shear stress and chick embryo transplantation) have not solved the issue of large blood vessel anastomosis. This may directly lead to delayed glomerular development and reduced filtration function, limiting the large-scale application of kidney organoids in drug testing and regenerative medicine.

To further enhance the functional maturity of kidney organoids, researchers have focused on augmenting the synergy between metabolism and mechanical forces. Metabolic induction strategies include stage-specific hypoxia treatment (to simulate the oxygen gradient in the kidney medulla) and sequential hormone additions (such as aldosterone or parathyroid hormone) to activate ion transport pathways in renal tubules [16]. Mechanical force stimulation, through bioreactors simulating glomerular capillary hemodynamics, has been shown to upregulate the expression of podocyte slit diaphragm protein (nephrin) and enhance filtration function in response to periodic fluid pressure [29]. The issue of long-term functional maintenance is particularly prominent. For instance, the epithelial cells of kidney organoids mature only to a mid- to late-fetal stage, which may affect various physiological functions, including transport [15]. Recently, Shi et al. developed an hPSC differentiation system that attempts to address the CD (collecting duct) deficiency in human kidney organoids by assembling induced renal mesenchyme with ureteric bud (UB) progenitor cells [37]. Additionally, other researchers have attempted to improve the incomplete simulation of the human kidney microenvironment in organoids through metabolic reprogramming strategies, such as sequential addition of hypoxia-sensitive factors to simulate the oxygen gradient in the renal medulla and hormone pulse stimulation to enhance urea concentration ability. However, maturity defects still limit their physiological relevance [32]. These engineering strategies collectively aim to construct a more physiologically relevant microenvironment for kidney organoids.

### 2.3. Standardization Breakthrough in Quality Assessment Technologies

Concurrently, robust and standardized methods to evaluate the quality and fidelity of the resulting organoids are equally critical for translational research. Innovations in quality assessment technologies have enhanced the reliability and application value of kidney organoids. Single-cell transcriptomics have revealed that early kidney organoids still face challenges with cell type fidelity (Table 1). For instance, early organoids often contain off-target neuronal-like cells, but optimized differentiation protocols have improved the proportion of target kidney cells to over 90% [38,39]. Recently developed high-content imaging technologies, combined with deep learning algorithms, have enabled automated quantitative analysis of organoid morphology (such as glomerular diameter and number of renal tubule branches) and functional maturity (such as fluorescence-labeled albumin uptake efficiency), providing standardized tools for high-throughput drug screening [9,40]. Furthermore, the standardized production of kidney organoids is limited by cell heterogeneity between batches and fluctuations in the composition of matrix gels [41,42]. Innovations in biomaterials provide new approaches to address this issue. Light-patterned hydrogels dynamically regulate morphogen gradients (such as BMP7/WNT), significantly improving the accuracy of nephron spatial arrangement and reducing structural variation [32]. However, despite engineering innovations like light-patterned hydrogels that improve the spatial arrangement precision of kidney organoids, standardized production still faces deep bottlenecks, such as insufficient vascular integration and difficulties in maintaining long-term functionality.

Overall, from a technological standpoint, kidney organoid technology is advancing toward clinical translation through interdisciplinary integration. However, maintaining long-term functionality, integrating across nephron segments, and achieving scalable production remain key challenges that need to be overcome.

## 3. Core Applications of Kidney Organoids

### 3.1. Disease Modeling

**Figure 1:**
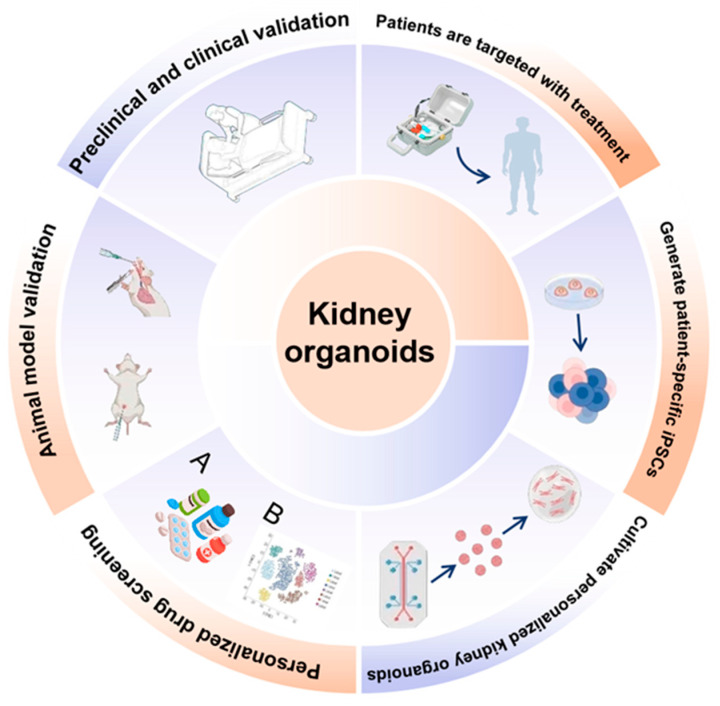
**Applications and translational framework of kidney organoids.** Schematic overview illustrating the generation, applications, and clinical translation of kidney organoids. Human pluripotent stem cells or patient-specific induced pluripotent stem cells (iPSCs) are differentiated into kidney organoids that recapitulate key structural and cellular features of the human kidney. These organoids enable personalized drug screening and toxicity testing, disease modeling, and mechanistic studies at the molecular and cellular levels. Kidney organoids are integrated with omics-based analyses (**A**,**B**), microfluidic and vascularized platforms, and functional assays to assess development, injury, and repair. Validation across animal models and preclinical and clinical studies supports their use in translational research. Ultimately, kidney organoids facilitate patient-specific modeling, targeted therapeutic discovery, and precision medicine approaches, bridging basic research with clinical application.

As the technology for kidney organoid culture continues to mature, with breakthroughs in microenvironment and microvascularization, kidney organoids have demonstrated significant advantages in the field of disease modeling (Figure 1). The application of kidney organoids in modeling various kidney diseases is becoming increasingly widespread. The selection of disease models discussed herein, which include diabetic kidney disease, genetic disorders, acute injury, polycystic kidney disease, and renal carcinoma, is deliberate because these conditions collectively represent major etiological categories of kidney disease such as metabolic, genetic, toxic, developmental, and neoplastic forms. This breadth demonstrates the capacity of organoid technology to address diverse disease mechanisms and to advance personalized medicine.

#### 3.1.1. Diabetic Kidney Disease (DKD)

In the field of diabetic kidney disease, the interaction between podocytes and mesangial matrix is a clear advantage of organoids over traditional cell models. Relevant studies show that the diabetic microenvironment induces molecular alterations such as the upregulation of angiotensin-converting enzyme 2 (ACE2) expression in renal tubular cells. This demonstrates the utility of kidney organoids in modeling diabetes-specific molecular pathology [45]. This model not only reveals the complex interaction between diabetes and viral infection but also highlights the unique advantage of kidney organoids in simulating the pathogenesis of multiple factor disease co-morbidity. Additionally, Xu Peng and colleagues used this model to discover that podocyte Golgi membrane protein 1 (GOLM1) was abnormally expressed under high-glucose stimulation. Through the GOLM1-EGFR-PPARγ-AMPKα signaling axis, inflammation and oxidative damage were exacerbated, a mechanism that was validated using a podocyte-specific gene knockout mouse model [49]. This work marks the deepening of organoid models from phenotypic replication to mechanistic exploration, which can accurately reveal the pathogenic pathway of specific genes in a complex microenvironment. Due to advancements in engineered microenvironments, Clerkin et al. utilized TGFβ-induced diabetic kidney disease injury models within kidney organoids to study the impact of mechanical environments on the propagation of early fibrosis-like features in DKD. The latest findings show that after TGFβ1 treatment, growth in a softer matrix was shown to reduce the expression of pSMAD3, thereby improving the expression of myofibroblast markers, such as α-smooth muscle actin (α-SMA) [33]. The relevance of these DKD models is often validated by quantifying the expression of podocyte injury markers (e.g., synaptopodin loss), fibrosis-related genes (e.g., COL1A1, FN1), and the uptake of albumin or specific dyes to assess proximal tubule function. These models complement clinical cohort studies, such as the DKD risk prediction model developed by Chang Baocheng’s team, which integrates nine indicators including age and UACR. High-risk stratification (≥16 points) was significantly associated with the increase in early injury marker KIM-1 detected in organoids, providing theoretical support for personalized interventions [50]. The higher physiological relevance of kidney organoid-microenvironment allows researchers to deeply analyze key pathological mechanisms that cannot be fully expressed in simple high-glucose 2D cultures. Its core value lies in reconstructing the “cell behavior–molecular network–microenvironment” triad, which drives disease occurrence and development, providing an irreplaceable research tool for uncovering the core pathological processes in diabetic kidney disease (Table 2).

#### 3.1.2. Genetic Kidney Diseases

In addition to metabolic diseases, the introduction of gene editing technologies in the study of genetic kidney diseases has further expanded the application boundaries of organoids. Unlike the microenvironment interventions used in diabetic kidney disease models, the modeling of genetic kidney diseases focuses more on gene editing. For example, human pluripotent stem cells with mutations in NPHS1 (encoding nephrin) or COL4A5 (associated with Alport syndrome) have been generated using CRISPR-Cas9. The organoids derived from these cells reproduce podocyte slit diaphragm deficiency or abnormal type IV collagen structure in the basement membrane, providing a platform for studying the correlation between genotype and phenotype [51]. This correlation is validated at the molecular level, for instance, by confirming the absence or mislocalization of nephrin in NPHS1-mutant organoids, or the aberrant assembly of collagen IV in models of Alport syndrome. Beyond phenotypic reproduction, the core value of genetically modified kidney organoids lies in their ability to provide a dynamic platform for deciphering the cellular and molecular mechanisms underlying disease development. This directly aids in the exploration of therapeutic strategies. Detailed mechanistic analysis benefits from the organoids’ ability to observe dynamic changes in cellular behavior within a near-physiological three-dimensional structure, which is difficult to achieve in traditional models. Gene editing has evolved kidney organoids from mere disease replicators into decoders of disease mechanisms and experimental fields for therapy. By elucidating the complete chain from genetic defects to cellular dysfunction and tissue pathological phenotypes, this technology has paved an irreplaceable path for understanding the nature of diseases and developing targeted intervention strategies.

#### 3.1.3. Acute Kidney Injury

With their three-dimensional structure and cellular heterogeneity, organoids have demonstrated pathological sensitivity beyond that of traditional 2D models in acute kidney injury (AKI) modeling. For example, AKI models have been induced in kidney organoids via ischemia-reperfusion or nephrotoxic drugs such as cisplatin. Studies show that cisplatin-treated kidney organoids exhibit upregulation of apoptosis markers BAX/BAK in proximal tubular cells and detachment of the brush border, with the severity of damage positively correlating with clinical dosage, which better mirrors the dose–response trends observed in clinical settings [44]. Functional validation of AKI in organoids includes measuring the upregulation of injury biomarkers (e.g., KIM-1, NGAL) and a decline in transepithelial electrical resistance, confirming both molecular and functional damage. This demonstrates that kidney organoids, as AKI models, have greater stability and structural diversity compared to traditional cell models as they more comprehensively replicate the kidney’s structure and intercellular communication.

#### 3.1.4. Polycystic Kidney Disease

In addition to common acute and chronic kidney diseases, the fine-tuning of kidney organoid microenvironments can also be used to model kidney cysts. Cruz et al. discovered that systematic replacement of physical components in kidney organoids affects cyst formation, highlighting the importance of the microenvironment in simulating polycystic kidney disease (PKD) in organoids. By adjusting the organoid microenvironment, it is possible to more accurately replicate the early phenotypic features of the disease [43]. In 2022, Xu et al. utilized tubule-like organoids to model autosomal dominant polycystic kidney disease (ADPKD). Cyst formation in these organoids successfully reconstructed the disease’s phenotypic characteristics [47]. These phenotypic changes are supported by molecular metrics, including the downregulation of polycystin proteins and upregulation of cyst-associated pathways (e.g., cAMP), which serve as key validation endpoints for drug screening. The following year, Mae et al. advanced the development of tubule-like organoids by establishing an expansion culture of hiPSC-derived ureteric bud tip cells (embryonic precursors that generate renal tubules and collecting ducts). They demonstrated that all CD (collecting duct) organoids derived from PKD1 hiPSCs spontaneously developed multiple cysts, elucidating the initiation mechanism of cyst formation [46]. Later, Vishy et al. used base-editing technology to construct organoids carrying the PKD1-R2430X nonsense mutation, successfully simulating the spontaneous cyst formation process. This model can be further used for drug screening, such as evaluating the efficacy of eukaryotic ribosomal selective glycosides (ERSGs), which restore polycystin expression by reading through the nonsense mutation, significantly inhibiting cyst expansion [48].

#### 3.1.5. Renal Carcinoma

In parallel, the development of kidney organoid technology has also advanced renal carcinoma research to some extent. For example, patient-derived clear cell carcinoma organoids successfully retained key molecular characteristics of the primary tumor, such as VHL deletion and persistent high expression of HIF-2α, and reproduced tumor heterogeneity. This model has shown significant value in drug screening [52]. The fidelity of such tumor organoids is validated by maintaining the expression of original tumor drivers (e.g., VHL loss, HIF-2α stabilization), histological features, and patient-specific drug response profiles in vitro. Additionally, Li et al. cultured 33 renal carcinoma organoid lines derived from common renal carcinoma subtypes, demonstrating good tumor microenvironment heterogeneity and providing an efficient platform for in vitro drug screening [53]. Zhang et al.’s latest research conducted CAR-T cell cytotoxicity assays in the developed ccRCC organoid model to assess the efficacy of targeting CD70 for treating ccRCC in vitro, further proving the significant role of kidney organoids in this field [54]. More importantly, the renal cancer organoids derived from these patients not only reproduce the genetic characteristics of the original tumor but also simulate the tumor microenvironment in vivo including immune cell infiltration and extracellular matrix components, providing a more realistic in vitro model for the study of tumor–stromal interaction and immunotherapy.

Kidney organoid technology, with its ability to highly simulate the microstructure of kidney units (glomeruli, renal tubules, and blood vessels) and intercellular interaction networks, has become a universal platform for constructing models of various kidney diseases, including metabolic diseases, genetic disorders, acute injuries, and tumors. Its core value lies in the successful simulation of key pathological phenotypes, such as high-glucose-induced podocyte injury, collagen metabolism disorders, and cyst formation, while pinpointing the relevant signaling pathways. Additionally, on the technological front, the integration of gene editing and microenvironment regulation helps reveal disease mechanisms, and at the clinical translation level, it facilitates the linkage of risk biomarkers to drug response validation. Through its unique advantages, kidney organoids provide a solid foundation for the development of personalized diagnostic and therapeutic strategies. However, organoid models still share a fundamental limitation: the absence of a functional immune microenvironment. The lack of immune cells restricts their ability to simulate critical pathological processes such as inflammation and fibrosis—hallmark features of many kidney diseases. Therefore, introducing immunological components (such as patient-derived immune cells or iPSC-derived macrophages) into existing models to reconstruct a more complete disease microenvironment has become a cutting-edge research direction.

#### 3.1.6. Chronic Kidney Disease (CKD) and Fibrosis

Beyond acute injury and cystic diseases, modeling the progressive nature of chronic kidney disease (CKD), particularly the culminating process of renal fibrosis, represents a significant frontier for kidney organoid technology. Leading research in the field has demonstrated that repetitive injury to kidney organoids can lead to the expansion of interstitial fibroblasts with myofibroblast characteristics, thereby modeling renal fibrosis in vitro [55]. Recent studies have established more specific modeling strategies. For instance, prolonged stimulation with exogenous transforming growth factor-β1 (TGF-β1) can successfully induce the production of extracellular matrix components, such as collagen, and upregulate key fibrosis markers like alpha-smooth muscle actin (α-SMA) in human induced pluripotent stem cell (iPSC)-derived kidney organoids [56]. These models not only recapitulate fibrotic phenotypes but are also utilized for pathological mechanism exploration and drug screening. For example, research using this model has revealed that bile acid receptor agonists can significantly attenuate TGF-β1-induced fibrosis by modulating downstream SMAD3 and TAZ pathways, providing clues for developing novel anti-fibrotic therapies [56]. Although challenges remain in replicating the complete temporal progression and functional decline of human CKD, these established CKD-organoid models provide an invaluable human-relevant platform for dissecting early fibrotic signaling pathways and screening novel anti-fibrotic compounds.

### 3.2. Kidney Organoids as Drug Development Platforms

In addition to disease modeling, kidney organoids have demonstrated multiple values in the field of drug development (Table 3). Compared to animal models, kidney organoids retain human-specific gene regulatory networks. Their core breakthrough lies in revolutionizing the kidney toxicity evaluation system by simulating the gene regulatory networks of mature kidneys, significantly enhancing the accuracy of toxicity predictions. Single-cell transcriptomic analysis shows that organoids can accurately model the complex gene regulatory networks present in mature kidneys. For example, data from the Yoshimura team validated the use of human kidney organoids to study normal developmental pathways and how pathogenic mutations leading to CAKUT or adult kidney diseases alter cell differentiation or cell functions [39]. This provides new insights for studying kidney development mechanisms and the impact of pathogenic mutations on cellular differentiation. The application of kidney organoids in autosomal recessive polycystic kidney disease further proves their enormous potential in drug testing [57]. Organoids also contribute to the development of novel drug delivery systems. For instance, research by DU et al. demonstrated that kidney organoids modified with EPO S/MAR DNA vectors allowed for the stable, long-term delivery of EPO, potentially enabling long-term, stable therapeutic protein delivery [20]. Organoids open new pathways for kidney-targeted drug delivery strategies, with their core advantage being the ability to overcome the limitation of human embryonic sample scarcity. They have, for the first time, enabled the long-term, dynamic, and visual observation of cell differentiation and function in human kidney development and disease progression. For example, by using reporter systems to track the fate determination of nephron progenitor cells in real-time, kidney organoids provide unprecedented direct human data for understanding developmental disorders and mutation effects.

### 3.3. Regenerative Medicine

As research continues to advance, the treatment options for kidney diseases have become increasingly diversified. However, existing treatments are still unable to fully prevent kidney damage from progressing to end-stage renal disease (ESRD). Kidney dialysis is inefficient, targeted drug treatments heavily rely on personalized medicine, and their effectiveness varies among individuals. Kidney transplantation faces the challenge of organ shortages. In this context, it is crucial to focus on in-depth research on kidney organoids. Some preliminary studies have been conducted, which revealed that kidney organoids subjected to high fluid shear stress during in vitro development showed significant enhancements in the abundance and maturity of the vascular systems within the tubular and glomerular compartments. Additionally, morphological changes in tubular epithelial cells and podocytes were observed. Although it remains uncertain whether the microvascular networks within these organoids are amenable to perfusion, the ability to enhance development through increased fluid flow in vitro has opened new pathways for studying organogenesis, nephrotoxicity, tubulointerstitial and glomerular diseases, as well as using simple microfluidic chips for kidney regeneration [29].

At the same time, the development of organoids has also contributed to the advancement of artificial kidneys. The integration of bio-artificial kidney devices requires solving issues related to the scalability of organoids and scaffold compatibility. Light-crosslinked porcine kidney decellularized matrix (KdMA) bio-ink, modified by methacrylation, provides a kidney-specific extracellular microenvironment. Its mechanical properties (0.67–4.81 kPa) match the stiffness of kidney tissue and support human embryonic kidney cells in forming >100 μm spheroids with over 90% survival rate [58]. Wang et al. successfully formed organized human-pig chimeric mesonephros structures in kidney-deficient pig embryos lacking SIX1 and SALL1, indicating the potential for generating humanized primitive organs in pigs with organogenesis disabilities [59]. Organoid chips have further pushed forward the functionalization of these systems. For example, applying fluid shear stress in microfluidic chips can enhance the creatinine clearance ability of organoids, simulating glomerular filtration function [51].

Although kidney organoid transplantation demonstrates regenerative potential, its clinical application still faces significant challenges. There are technical bottlenecks in large-scale production of high-quality, uniform organoids. Post-transplant, the effective integration of organoids with the host kidney at both anatomical and functional levels, particularly issues related to nerve innervation and urine drainage, remain unresolved. The risk of immune rejection from syngeneic or xenogeneic transplantation needs to be managed with engineered immune tolerance strategies. Most critically, the efficiency of vascularization post-transplantation is extremely low and uncontrollable. The lack of a functional vascular network for rapid anastomosis severely limits organoid survival and functional performance. Solving the vascularization issue is a key prerequisite for achieving successful transplantation.

## 4. Discussion

**Figure 2:**
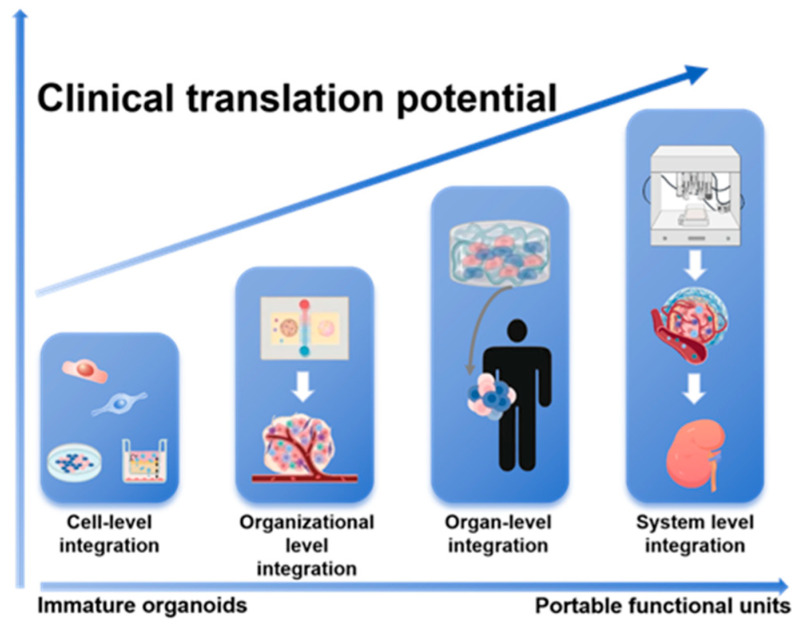
**A Hierarchical Integration Paradigm for Advancing Kidney Organoids toward Clinical Application.** This schematic outlines a staged engineering strategy to overcome limitations in organoid maturity and function. The paradigm progresses through sequential tiers of complexity—from cell-level and organizational-level integration to organ-level and ultimately system-level integration. This stepwise approach drives the transformation of immature organoids into functional, portable units with enhanced translational potential for regenerative medicine.

Kidney organoid technology, by simulating human kidney development and pathological microenvironments, provides an irreplaceable platform for disease mechanism analysis, drug screening, and regenerative medicine. Current research has achieved a significant leap from static structural biomimicry to dynamic functional simulation. For example, gene-edited organoids precisely reproduce the phenotypes of genetic kidney diseases, engineered vascular networks partially restore filtration function, and AI-driven quality control systems improve standardization levels (Figure 2) [60]. By integrating these technologies, we can envision “next-generation intelligent organoids” that respond to physiological signals for dynamic regulation. For example, a ‘fibrosis-sensing’ kidney organoid-on-a-chip could use biosensors to detect early extracellular matrix stiffening and trigger the on-demand release of anti-fibrotic drugs. Similarly, an intelligent nephrotoxicity screening platform could employ continuous imaging and effluent analysis of organoids, with machine learning algorithms correlating multiparametric data to predict human-relevant renal safety profiles with high accuracy.

Despite the significant progress made by kidney organoid technology in simulating kidney development and diseases, its clinical translation still faces deep challenges. The foremost among these are achieving long-term functional maturity, engineering perfusable and integrated vascular networks, and establishing scalable, standardized production processes within appropriate ethical frameworks. To realize its full therapeutic potential, future research must pivot towards engineering solutions that address vascularization as the central bottleneck, which is intrinsically linked to functional maturation. Concurrently, the deeper integration of bioengineering tools—such as organ-on-a-chip systems, AI-driven functional analytics, and biomaterials-mediated microenvironmental control—will be crucial for developing robust and predictive next-generation models. This interdisciplinary convergence will ultimately drive the field from foundational observation towards transformative clinical application in personalized medicine and regeneration.

## Figures and Tables

**Table 1:** **Timeline of Major Advances in Kidney Organoid Models.** This timeline summarizes key methodological breakthroughs and disease modeling applications in kidney organoid research from 2014 to 2025. The selected milestones represent pivotal studies that established foundational protocols, enabled new types of disease modeling, or addressed major challenges in maturation and scalability. The progression illustrates the field’s evolution from in vitro nephron generation toward functional assessment and clinical translation.

Year	Milestone Achievement	Significance	Key References
2014–2015	First generation of kidney organoids from human pluripotent stem cells (hPSCs) that contain multiple nephron segments.	Established the foundational protocol; demonstrated the principle of mimicking nephrogenesis in vitro.	[15,22]
2017	Modeling polycystic kidney disease (PKD): using patient-specific organoids and identifying microenvironmental cues critical for cystogenesis.	Provided the first proof-of-concept for using organoids to model a complex genetic kidney disease and study its mechanism.	[43]
2019	Enhanced vascularization and maturation: via flow-induced mechanical stimulation in microfluidic chips.	Introduced bioengineering tools (organ-on-a-chip) to significantly improve organoid maturity and vascular network formation.	[29,34]
2020–2022	Advanced disease modeling: for diabetic kidney disease (DKD) and acute kidney injury (AKI), linking in vitro phenotypes to clinical biomarkers.	Demonstrated the utility of organoids in modeling acquired diseases and validating clinical correlations (e.g., drug toxicity, KIM-1 biomarker).	[44,45]
2022–2023	Precise genetic disease modeling: using gene-edited and patient-derived iPSCs for Alport syndrome and ADPKD.	Enabled isogenic comparisons and deep mechanistic studies of genetic disorders, advancing towards personalized medicine.	[46,47]
2023–2024	Single-cell multi-omics and deep learning: for standardized quality control and profiling of organoid heterogeneity.	Established new standards for objective assessment and revealed cell-type-specific responses, enabling high-throughput screening.	[28,39]
2024–2025	Functional maturation & drug discovery: Generation of long-term expandable nephron progenitors and application in compound screening (e.g., for ADPKD).	Addressed long-term culture and scalability challenges, directly applying organoids to identify therapeutic candidates.	[33,48]
2025	Towards clinical translation: Focus on integrated vascularization, immune microenvironment incorporation, and in vivo functional assessment.	Represent the current frontier aimed at overcoming the final barriers to regenerative medicine applications.	[20,37]

**Table 2:** **Kidney Disease Models in Organoid Platforms.** This table demonstrates the capability of human pluripotent stem cell-derived and patient-derived kidney organoids to recapitulate key pathological features across a spectrum of kidney diseases. By employing strategies such as gene editing, microenvironmental tuning, and chronic factor exposure, these in vitro models faithfully mimic human-specific disease phenotypes, ranging from structural defects in genetic disorders to functional injury and fibrotic progression. They serve as essential platforms for mechanistic dissection and therapeutic exploration.

Disease Category	Pathological Feature	Organoid Model/Strategy	Key Findings
Diabetic Kidney Disease	Hyperglycemia-induced podocyte injury, oxidative stress, fibrosis	hiPSC-derived organoids under high-glucose conditions or TGF-β1 treatment; microenvironment tuning (e.g., matrix stiffness).	Recapitulated GOLM1-EGFR-PPARγ-AMPKα axis exacerbating injury; softer matrices reduced TGF-β-induced fibrotic markers.
Genetic Kidney Diseases (e.g., Alport Syndrome)	Podocyte slit diaphragm defects, abnormal glomerular basement membrane (type IV collagen)	CRISPR-Cas9 gene-edited hPSCs (e.g., NPHS1, COL4A5 mutations) differentiated into organoids.	Reproduced genotype-specific structural defects; platform for genotype-phenotype correlation and mechanistic dissection.
Acute Kidney Injury (AKI)	Tubular cell apoptosis, brush border loss, biomarker (e.g., KIM-1) upregulation	Chemical induction (e.g., cisplatin) or ischemia-reperfusion modeling in hPSC-derived organoids.	Dose-dependent injury response mirroring clinical trends; superior pathological sensitivity vs. 2D models.
Polycystic Kidney Disease (PKD)	Spontaneous cyst formation, tubular dilation	hiPSC-derived ureteric bud/collecting duct organoids from PKD1-mutant cells; base-edited organoids (e.g., PKD1-R2430X).	Elucidated cyst initiation mechanisms; platform for drug screening (e.g., read-through compounds).
Renal Carcinoma (e.g., ccRCC)	Tumor heterogeneity, key mutation retention (e.g., VHL), tumor microenvironment	Patient-derived tumor organoids (PDTOs) from surgical samples; co-culture models (e.g., with CAR-T cells).	Retained original tumor genetics and drug response profiles; useful for immunotherapy efficacy testing.
Chronic Kidney Disease/Fibrosis	Progressive fibrosis, myofibroblast activation, ECM deposition	Chronic TGF-β stimulation, metabolite accumulation models in long-term organoid cultures.	Models key aspects of fibrotic progression; used for testing anti-fibrotic therapies.

**Table 3:** **Applications of Kidney Organoid Models in Drug Discovery and Assessment.** This table exemplifies the utility of human kidney organoid models in various stages of drug discovery and toxicity assessment. The listed studies showcase applications ranging from high-throughput screening and drug repurposing to the validation of clinical candidates and nephrotoxicity evaluation. These models provide a physiologically relevant human platform that recapitulates disease-specific phenotypes, bridging the gap between traditional in vitro assays and clinical trials for renal therapeutics.

Drug/Compound Name	Stage/Purpose	Kidney Organoid Model Used	Key Findings/Progress
Tamibarotene	Phase IIa Clinical Trial	iPSC-derived collecting duct organoids (ADPKD cyst model)	Identified via screening in ADPKD patient iPSC-derived cyst organoids. This retinoic acid receptor agonist significantly inhibited cystogenesis and has entered a Phase IIa trial for ADPKD.
Minoxidil	Preclinical validation & drug repurposing	iPSC-derived polycystic kidney disease organoids	Revealed a link between cystogenesis and autophagy defects. Validated the FDA-approved drug minoxidil as an autophagy activator, effectively attenuating cyst formation both in vitro and in vivo.
Tolvaptan	Efficacy validation of an approved drug	iPSC-derived collecting duct organoids (ADPKD cyst model)	Validated the cyst-inhibiting effect of tolvaptan (the only clinically approved drug for ADPKD) in this human organoid model, confirming the platform’s reliability for efficacy assessment.
Imatinib	Drug repurposing/nephroprotective agent	iPSC-derived kidney organoids (cisplatin injury model)	Established an automated high-throughput screening platform using kidney organoids. Screened an FDA-approved drug library and identified imatinib’s potential protective effect against cisplatin-induced acute kidney injury.
Doxorubicin	Nephrotoxicity assessment	hiPSC-derived functional kidney organoids (3D-bioprinted)	Demonstrated that the bioprinted kidney organoids recapitulated doxorubicin-induced injury (e.g., upregulation of injury marker KIM-1), validating their utility for nephrotoxicity testing.
(Various compound libraries)	High-throughput nephrotoxicity/therapeutic screening	iPSC-derived kidney organoids	Established a screening pipeline integrating automated 3D imaging and machine learning analysis of kidney organoids for large-scale compound evaluation of nephrotoxicity and therapeutic effects.
Fibrolisine (candidate)	Preclinical candidate evaluation	Advanced human kidney fibrosis organoid	The EU-funded FibroTarg project utilizes a sophisticated human kidney fibrosis organoid model to evaluate the efficacy of its first-in-class anti-fibrotic candidate, Fibrolisine.

## Data Availability

No new data were created or analyzed in this study. Data sharing is not applicable to this article.

## Abbreviations

**Abbreviation**	**Full Term**
ACE2	Angiotensin-converting enzyme 2
AKI	Acute Kidney Injury
ADPKD	Autosomal Dominant Polycystic Kidney Disease
CKD	Chronic Kidney Disease
DKD	Diabetic Kidney Disease
ECM	Extracellular Matrix
GelMA	Gelatin Methacryloyl
hPSC	Human Pluripotent Stem Cell
iPSC	Induced Pluripotent Stem Cell
PKD	Polycystic Kidney Disease
TGF-β	Transforming Growth Factor Beta
